# MarR family proteins sense sulfane sulfur in bacteria

**DOI:** 10.1002/mlf2.12109

**Published:** 2024-05-15

**Authors:** Guanhua Xuan, Luying Xun, Yongzhen Xia

**Affiliations:** ^1^ State Key Laboratory of Microbial Technology Shandong University Qingdao China; ^2^ State Key Laboratory of Marine Food Processing & Safety Control Ocean University of China Qingdao China; ^3^ School of Molecular Biosciences Washington State University Pullman Washington USA

**Keywords:** cysteine thiols, disulfide bond, MarR protein family, ROS, sulfane sulfur

## Abstract

Members of the multiple antibiotic resistance regulator (MarR) protein family are ubiquitous in bacteria and play critical roles in regulating cellular metabolism and antibiotic resistance. MarR family proteins function as repressors, and their interactions with modulators induce the expression of controlled genes. The previously characterized modulators are insufficient to explain the activities of certain MarR family proteins. However, recently, several MarR family proteins have been reported to sense sulfane sulfur, including zero‐valent sulfur, persulfide (R‐SSH), and polysulfide (R‐SnH, *n* ≥ 2). Sulfane sulfur is a common cellular component in bacteria whose levels vary during bacterial growth. The changing levels of sulfane sulfur affect the expression of many MarR‐controlled genes. Sulfane sulfur reacts with the cysteine thiols of MarR family proteins, causing the formation of protein thiol persulfide, disulfide bonds, and other modifications. Several MarR family proteins that respond to reactive oxygen species (ROS) also sense sulfane sulfur, as both sulfane sulfur and ROS induce the formation of disulfide bonds. This review focused on MarR family proteins that sense sulfane sulfur. However, the sensing mechanisms reviewed here may also apply to other proteins that detect sulfane sulfur, which is emerging as a modulator of gene regulation.

## INTRODUCTION

**Figure 1:**
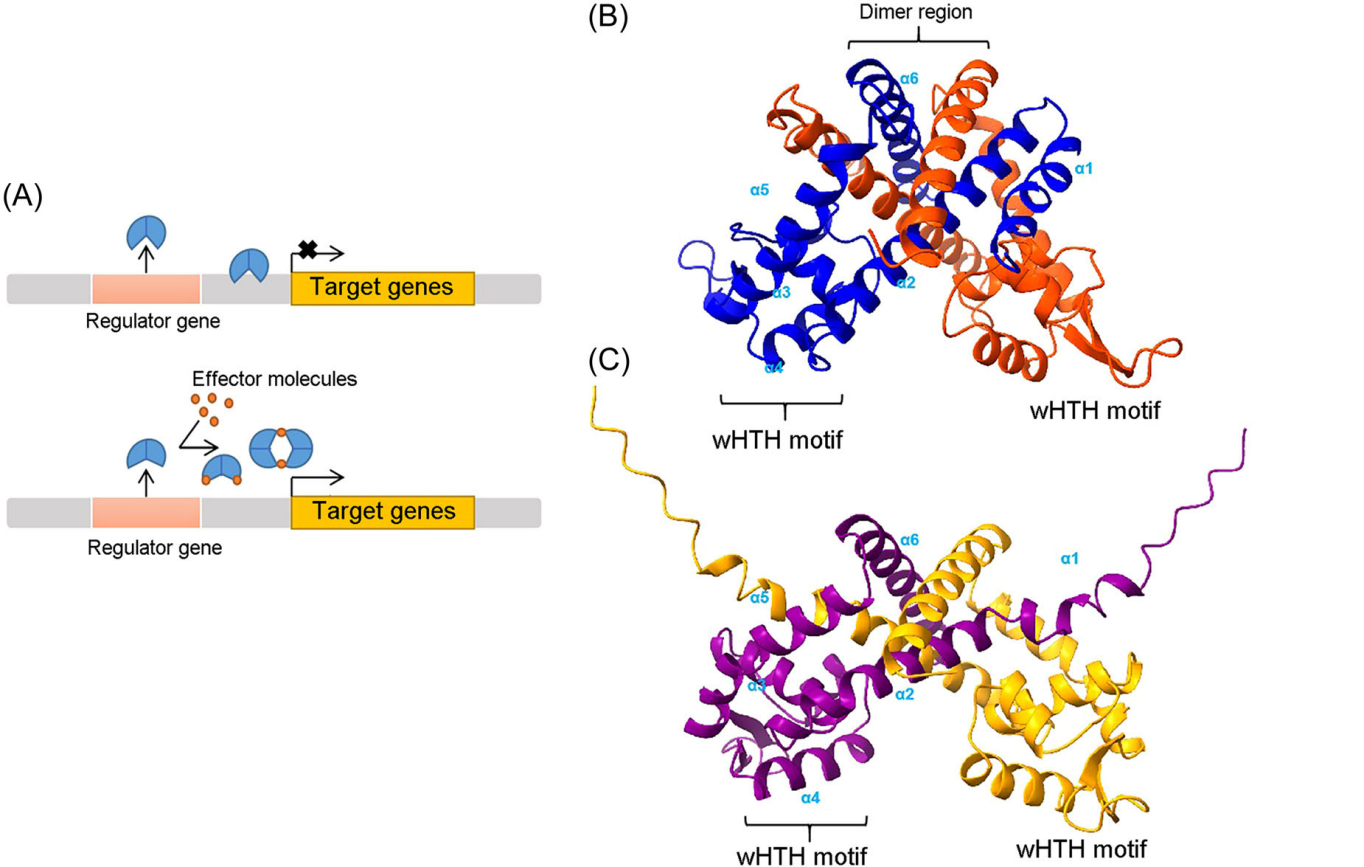
A typical mode of gene regulation by multiple antibiotic resistance regulator (MarR) family proteins. (A) In the absence of modulators, the MarR family protein (shown in blue) binds to a specific sequence within its target operator and represses the transcription of the targeted genes. Upon binding modulators, the MarR family protein undergoes a conformational change that results in the depression of its controlled genes. AlphaFold2 was used to predict the structures of *Liberibacter asiaticus* LdtR (B) and *Pseudomonas aeruginosa* OspR (C). Each subunit of the MarR dimer consists of six α‐helices, of which a winged helix‐turn‐helix (wHTH) motif is responsible for DNA binding. The protein structures were generated using ChimeraX^11^, ^12^.

The multiple antibiotic‐resistance regulator (MarR) was first identified in *Escherichia coli* ^1^, ^2^. It controls the transcription of genes that code for antibiotic resistance. Subsequent studies have revealed that MarR homologs are common in bacteria and are involved in the control of different processes, including adhesion, virulence, environmental stress resistance, and antibiotic resistance^3^, ^4^, ^5^. Sequenced bacterial genomes have approximately seven MarR paralogs per genome on average, but only a few of the predicted MarR homologs have been experimentally characterized^4^. These homologs are grouped into the MarR family of transcription factors. The crystal structures of several MarR family proteins have been reported, including MexR from *Pseudomonas aeruginosa* ^6^, MarR from *Escherichia coli* ^7^, and SlyA from *Enterococcus faecalis* ^8^. MarR family proteins usually function as dimers, and each subunit consists of a DNA‐binding domain and a dimerization region, which is formed by an interdigitation of N‐ and C‐terminal helices α1, α5, and α6 from both subunits^4^. MarR family proteins often show a similar organization of genetic loci with regulator genes and target genes that are usually divergently oriented. Upon binding to modulator molecules or sensing environmental signals, the conformation of the MarR homodimer changes, consequently resulting in the dissociation of the repressor from the DNA‐binding site and induction of gene expression^4^, ^9^, ^10^ (Figure 1).

Small‐molecule ligands, metals, and reactive oxygen species (ROS) have been identified as the modulators of MarR family proteins. As the proteins do not have a specific modulator‐binding domain, the dimerization interface is usually involved in binding small organic modulators^13^, ^14^. The response to metals and ROS may involve one or more amino acid residues, usually cysteine residues. Most reported MarR family proteins have at least one cysteine residue^15^, which often participates in ROS sensing, such as OhrR^16^ and BifR^17^. MarR family proteins that do not contain cysteine residues may not sense ROS but sense small ligands. For example, *Burkholderia thailandensis* MftR responds to urate, activating the genes involved in urate catabolism^18^, and *Streptomyces coelicolor* PcaV responds to the anionic phenolic ligand protocatechuate, inducing the expression of the genes that encode the enzymes involved in the β‐ketoadipate pathway of catechol catabolism^19^. Both bind the ligands between the dimerization and DNA‐binding regions.

ROS‐responding MarR regulators may also sense sulfane sulfur, a common cellular component in most cells. Sulfane sulfur refers to a sulfur atom with zero valence linked to one or two sulfur atoms, including octasulfur (S_8_), inorganic polysulfide (H_2_S_*n*_, *n* ≥ 2), and organic polysulfide (RS_*n*_H, *n* ≥ 2)^20^, ^21^. These reactive sulfur species are emerging as new cellular components that plays important roles in cell signaling, redox homeostasis, and metabolic regulation^22^. Recently, several ROS‐responding MarR regulators, including MgrA^23^, MexR^24^, MarR^25^, and OhrR^26^, have been shown to sense sulfane sulfur owing to their Cys residues^27^, ^28^, ^29^.

In this review, we highlight recent research studies on MarR family proteins that respond to different cues, especially the newly discovered sulfane sulfur, which may have the potential as a common signaling cue in bacteria.

## PRODUCTION AND METABOLISM OF CELLULAR SULFANE SULFUR

Zero‐valent sulfur is produced via several cellular pathways and spontaneously reacts with cellular thiols and hydrogen sulfide (H_2_S) to form RS_*n*_H and H_2_S_*n*_ ^30^. These sulfur species are collectively referred to as sulfane sulfur, which is produced by several enzymes from cystine, cysteine, and H_2_S. Cystathionine β‐synthase and cystathionine γ‐lyase generate sulfane sulfur from cystine^31^. 3‐Mercaptopyruvate sulfurtransferase and cysteinyl‐tRNA synthetase 2 produce sulfane sulfur from cysteine^20^, ^32^. Sulfide‐quinone oxidoreductase (SQR) oxidizes H_2_S into sulfane sulfur^33^. Owing to the potential toxicity of sulfane sulfur at high concentrations, cellular mechanisms exist for the regulation of its level. Excessive sulfane sulfur can be either oxidized by persulfide dioxygenase (PDO) into sulfite or reduced by cellular thiols, thioredoxin, and glutaredoxin into H_2_S^33^, ^34^, ^35^. Mammals exhibit significant levels (>100 μM) of sulfane sulfur in their plasma, cells, and tissues^31^. Ran et al.^36^ showed that sulfane sulfur content varies with the growth phase in both bacterial and cell line cultures, indicating that sulfane sulfur may be an important signal that mediates many physiological and pathologic processes.

## MODULATION OF MarR FAMILY PROTEINS VIA THE BINDING OF SMALL ORGANIC LIGANDS

For MarR family proteins, the site for organic ligand binding is usually at the junction between the dimerization and DNA‐binding domains^13^. The junction may bind salicylate, the aminoglycoside antibiotic kanamycin, and the aminoglycoside antibiotic streptomycin^37^. The binding of antibiotics is consistent with their common functions, regulating the resistance to antibiotics. A structural analysis of SAR2349 from *Staphylococcus aureus* revealed that it binds four salicylates: three at the junction between the DNA‐binding and dimerization domains and one near the DNA‐binding domain. SAR2349 can also simultaneously bind salicylate and kanamycin. Similar binding of multiple ligands has also been reported for TcaR from *Staphylococcus epidermidis* ^37^, ^38^. The nonspecific binding is likely due to the limitation of the junction between the dimerization and DNA‐binding domains, which has not specifically evolved to bind ligands. The junction contains a hydrophobic cavity that may weakly interact with different hydrophobic ligands^37^. Structural analyses of MTH313 and ST1710^39^, ^40^, MarR family proteins from *Methanobacterium thermoautotrophicum* and *Sulfolobus tokodaii*, respectively, provide evidence for the displacement of the DNA‐binding helix upon ligand binding, which hinders DNA binding.

Some identified ligands may not be physiologically relevant because of their low affinity^41^. *E. coli* MarR has been cocrystallized with salicylate only at a high salicylate concentration (approximately 5 mM), corresponding to the reported induction of MarR‐repressed genes only at high salicylate concentrations in whole‐cell studies^42^, ^43^. As high salicylate concentrations are not associated with antibiotics, salicylate is unlikely to be a physiological modulator of MarR in *E. coli*.

## REGULATION OF MarR FAMILY PROTEINS BY ROS

ROS are commonly generated via aerobic respiration or the reaction between O_2_ and univalent electron donors in all organisms that grow under aerobic conditions. ROS can induce the covalent modifications of biological molecules, including DNA, protein, and lipids, resulting in the loss or gain of functions^44^, ^45^. Several MarR family proteins are known to respond to ROS. *Bacillus thailandensis* BifR, a redox‐sensitive repressor of genes involved in biofilm formation and antibiotic synthesis, forms a cross‐linked dimer upon the addition of H_2_O_2_, and the oxidized BifR competes more effectively with RNA polymerase for DNA binding, further repressing its controlled genes^17^. *Corynebacterium glutamicum* CosR senses peroxide stress with the formation of interprotomer disulfide bonds, which leads to the depression of the target genes and increased resistance to oxidative stress^46^. OhrR is an organic peroxide‐sensing repressor that regulates the peroxidase gene *ohr*. OhrR mainly responds to host‐derived organic hydroperoxides, allowing *ohr* transcription, and Ohr is able to remove organic hydroperoxides^47^, ^48^. In addition, Ohr has moderate activities toward peroxynitrite and H_2_O_2_ ^49^, ^50^. OhrR homologs may have different preferred inducers. *Bacillus subtilis* OhrR^51^, ^52^ is greatly induced by organic peroxide rather than by NaClO. *Xanthomonas campestris* OhrR^53^, ^54^ is more sensitive to complex organic peroxides such as linoleic acid hydroperoxide rather than to H_2_O_2_, and *Agrobacterium tumefaciens* OhrR preferentially senses less‐complex organic peroxides such as cumene hydroperoxide^55^. *Shewanella oneidensis* OhrR MR‐1 can be induced by H_2_O_2_ and organic hydroperoxides^56^. Thus, ROS is a common modulator of MarR family proteins.

MarR family regulators often use cysteine residues for ROS sensing, such as *B. subtilis* OhrR and *S. aureus* SarZ, which belong to the 1‐Cys‐type MarR/OhrR subfamily^47^, ^57^. The initial step involves the oxidation of the sensing cysteine to a sulfenic acid (C‐SOH) that still retains DNA‐binding activity. The oxidized cysteine could further react with bacillithiol (BSH) or benzene thiol to produce a mixed disulfide bond such as S‐bacillithiolated OhrR^52^ or S‐thiolated SarZ^57^, rendering the protein incapable of DNA binding. By contrast, 1‐Cys‐type *S. aureus* MgrA can be oxidized into Cys‐SOH form, which directly causes the dissociation of MgrA from the operator DNA^28^. However, most redox‐responsive MarR family proteins contain two or more cysteine residues, including *P. aeruginosa* MexR, *X. campestris* OhrR, and *P. aeruginosa* OhrR. For MexR, two redox‐active cysteines, Cys30 and Cys62, are located on helix α1 and in the loop between helices α3 and α4^6^. Upon sensing oxidative signals, including glutathione disulfide, hydrogen peroxide (H_2_O_2_), and cumene hydroperoxide, two interprotomer disulfide bonds, Cys30‐Cys62′ and Cys30′‐Cys62, are formed within a MexR dimer^29^. This oxidation induces the conformational change in the DNA‐binding domain, preventing the binding of MexR to the operator DNA, thus activating the expression of the *mexAB‐oprM* regulon, which encodes a multiple drug efflux pump for the resistance of antibiotics. The 2‐Cys‐type *X. campestris* OhrR is also endowed with a similar oxidant‐sensing mechanism, through which an interprotomer disulfide bond is formed between Cys22 and Cys127′ upon oxidation^48^. *P. aeruginosa* OhrR has been characterized to govern organic hydroperoxide sensing^58^. The N‐terminus Cys19 plays an important role in organic hydroperoxide sensing and is oxidized into Cys19‐SOH, which reacts with the conserved Cys121 at the C‐terminus to form a disulfide bond. The formation of the disulfide bond prevents further oxidation of the redox‐sensing Cys19. Both Cys19 and Cys121 are important in the overall sensing process. Despite the common use of cysteine residues, these MarR family proteins may have different sensing mechanisms.

## MODULATION OF MarR FAMILY PROTEINS BY METAL BINDING

Two zinc ion (Zn^2+^)‐binding MarR family proteins have been well studied. The adhesin competence regulator (AdcR) of *Streptococcus pneumoniae* is the first metal‐dependent member of the MarR family proteins to be characterized^59^. AdcR regulates the transcription of a Zn^2+^ uptake system and several surface‐attached proteins for surface adhesion^60^. The binding of Zn^2+^ to the metal‐binding pocket leads to a structural rearrangement that causes AdcR binding to the operator sequences of genes regulated by AdcR, resulting in the inhibition of their expression. Conversely, during zinc starvation, AdcR dissociates from the target DNA, allowing the derepression of the genes and zinc uptake. The crystal structure revealed that Zn^2+^ binds AdcR with high affinity at two distinct sites, one of which is a tetracoordinate site involving E24, H42, H108, and H112 as the primary sensing site^59^. The other Zn^2+^‐binding MarR protein is LdtR, a master regulator in *Liberibacter* *asiaticus* that is linked to the regulation of more than 180 genes^61^ involved in energy production, cell motility, cell wall envelope, and zinc uptake^62^. LdtR tightly binds Zn^2+^ with Cys28 and Thr43, the two key residues located near the junction between the dimerization and DNA‐binding domains. The binding results in a conformational change of LdtR, disrupting the binding of LdtR to the operator and inducing the expression of various genes^63^. Thus, the binding of Zn^2+^ leads to different outcomes for AdcR and LdtR.

*E. coli* MarR has been reported to respond to low Cu^2+^ levels^27^. Cu^2+^ may be released from the inner membrane‐bound copper proteins involved in aerobic respiration upon oxidative damage or exposure to antibiotics. The addition of 100 μM Cu^2+^ led to a significant increase in the expression of LacZ from the *marR*::*lacZ* reporter system. Cu^2+^ causes the oxidization of a cysteine residue (Cys80) on *E. coli* MarR to generate disulfide bonds between two MarR dimers, triggering the dissociation of MarR from its DNA‐binding site. MarR senses Cu^2+^ indirectly via the oxidation and formation of disulfide bonds instead of Cu^2+^ binding.

## EVIDENCE OF SULFANE SULFUR AS A NEW MODULATOR OF GENE REGULATORS

In recent years, the physiological functions of sulfane sulfur in bacteria have emerged. Several gene regulators such as *Cupriavidus pinatubonensis* FisR^64^, *Rhodobacter capsulatus* SqrR^65^, *S. aureus* CstR^66^, *E. coli* OxyR^67^, and *S. coelicolor* CsoR^68^ have been identified that directly respond to intracellular sulfane sulfur, thereby activating the transcription of sulfur‐oxidizing genes. Gene regulators unrelated to sulfur metabolism may also have the ability to sense sulfane sulfur. Sulfane sulfur posttranslationally modifies AdpA in *S. coelicolor* by forming a persulfide (Cys62‐SSH), which decreases the affinity of AdpA to its self‐promoter DNA and further activates the expression of genes related to actinorhodin biosynthesis^69^. LasR is a master quorum‐sensing regulator that responds to a homoserine lactone‐type autoinducer^70^. LasR activity is also controlled by cellular sulfane sulfur in *P. aeruginosa*. When LasR reacted with sulfane sulfur, a pentasulfur link between Cys201 and Cys203 is formed, which significantly enhances the activity of LasR as a transcription activator of the genes involved in quorum sensing and virulence^71^. When the cell culture enters the late stationary or decline phase, the cellular sulfane sulfur concentration decreases, which leads to decreased LasR activity despite sufficient amounts of its autoinducer. Furthermore, several MarR family proteins that regulate resistance to antibiotics and oxidative stress have been shown to respond to sulfane sulfur^27^, ^28^, ^29^.

The protein structures of SqrR and CstR after sensing sulfane sulfur have been elucidated, and sulfane sulfur induces the formation of a tetrasulfide link and disulfide bond between the cysteine residues, respectively^72^, ^73^. Although no structural analyses have been performed for the interaction of MarR family proteins with sulfane sulfur, their reaction mechanisms can be speculated by leveraging the structural information of SqrR and CstR^72^, ^73^. Continued efforts to elucidate the interaction between MarR family proteins and sulfane sulfur through structural biology approaches will reveal mechanistic insights into the posttranslational modification of MarR family proteins by sulfane sulfur.

## MODULATION OF MarR FAMILY PROTEINS BY SULFANE SULFUR

**Figure 2:**
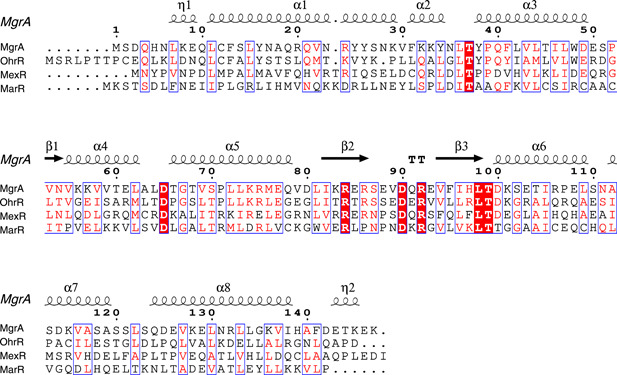
Comparison of the amino acid sequences of four MarR family proteins. MgrA (Uniprot: P0C1S0), OhrR (Uniprot: Q9HZZ4), MexR (Uniprot: P52003), and MarR (Uniprot: P27245) were used for multiple sequence alignment using Clustal Omega^74^. The outcomes were visualized using ESPript3.0^75^. The MgrA protein structure file (PDB: 2BV6) was referenced to obtain secondary structure information with ESPript3.0.

MgrA, OhrR, MexR, and MarR are four MarR family proteins that have been reported to sense sulfane sulfur^27^, ^28^, ^29^. Although they share similarities in structure, they exhibit significant diversities at the sequence level (Figure 2). The average similarity between MexR, MarR, OhrR, and MgrA does not exceed 35%. This variation in amino acid sequences may lead to each MarR family member recognizing different DNA targets and regulating distinct physiological functions; however, they all respond to sulfane sulfur.

### *E. coli* MarR

**Figure 3:**
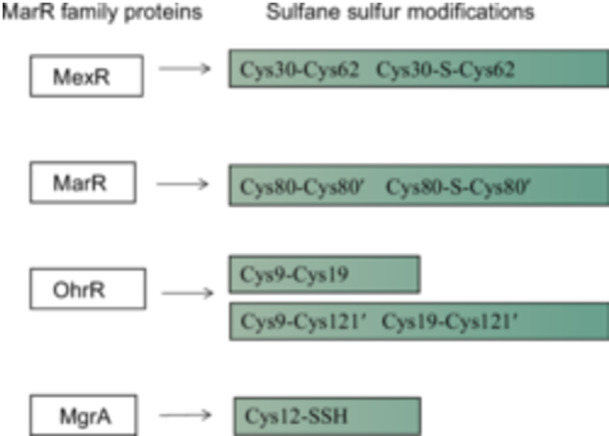
Schematic representations of the reported MarR family proteins that sense sulfane sulfur, forming various modifications on key cysteine residues.

*E. coli* MarR is the first member of the MarR family to be characterized. MarR regulates multiple antibiotic resistance operons *marRAB*, encoding itself (MarR); the global gene regulator MarA, which activates the expression of many genes involved in resistance to antibiotics^2^, ^43^, ^76^. In a recent report, sulfane sulfur was also reported to be a modulator of MarR. MarR senses elevated sulfane sulfur levels and mediates a dose‐dependent derepression of the repressed genes. Upon interaction with sulfane sulfur, MarR undergoes a process where it establishes either a disulfide bond (Cys80‐Cys80') or a trisulfide bond (Cys80‐S‐Cys80') connecting two dimers (Figure 3), resulting in the formation of a tetramer. The tetramer may form one disulfide bond or two disulfide bonds. Owing to the formation of disulfide and trisulfide bonds, MarR becomes unable to bind to its cognate DNA^25^. As sulfane sulfur levels in *E. coli* vary with the growth phase, reaching maximum levels at the late log and early stationary phases of growth^36^, corresponds to the expression of the *marRAB* operon that codes for an efflux pump of multiple antibiotics^25^.

### *P. aeruginosa* MexR

*P. aeruginosa* antibiotic resistance mechanisms include low outer membrane permeability, horizontal gene transfer, mutational changes, and the expression of multidrug efflux pumps^77^, ^78^. The MexAB‐OprM multidrug efflux pump is of particular interest because its overexpression is a major contributor to the multiple drug resistance of *P. aeruginosa* ^79^, ^80^. The *mexAB‐oprM* operon is regulated by the MarR family protein MexR, which binds to the intergenic region between *mexR* and *mexA*, repressing the transcription of *mexR* and *mexAB‐oprM* ^81^. Mutations in MexR or the conformational change in the MexR dimer would prevent the binding of MexR to its target DNA, which leads to the hyperexpression of MexAB‐OprM^82^. In a recent report published in *Molecular Microbiology*, Xuan et al^24^. described that the MexR activity is regulated by cellular sulfane sulfur, which leads to its dissociation from DNA, resulting in antibiotic resistance. MexR has been demonstrated to detect various sulfane sulfur species such as H_2_S_*n*_, S_8_, GSSH, and Cys‐SSH. Sulfane sulfur directly modifies MexR, forming disulfide and trisulfide links between Cys30 and Cys62 residues (Figure 3). The concentration of sulfane sulfur in *P. aeruginosa* varies with the growth phase. Elevated sulfane sulfur levels during the early stationary phase lead to a simultaneous increase in the expression of the *mex* operon. The observation offers a mechanistic explanation of how the bacterium induces the production of MexAB‐OprM at the stationary growth phase without antibiotic exposure^24^.

### *P. aeruginosa* OhrR

*P. aeruginosa* OhrR, a MarR family protein, also responds to sulfane sulfur. After reacting with sulfane sulfur, PaOhrR forms three disulfide bonds: an intraprotomer disulfide bond (Cys9–Cys19) and two interprotomer disulfide bonds (Cys9–Cys121′ and Cys19–Cys121′)^26^ (Figure 3). The formation of disulfide bonds reduces its binding to the target DNA, activating the *ohr* expression. Both ROS and sulfane sulfur sensing are involved in the formation of disulfide bonds in OhrR and the activation of *ohr* transcription^26^, ^58^, ^83^.

### *S. aureus* MgrA

MgrA, a member of the MarR protein family, governs the expression of around 350 genes^84^. Functioning as a global regulator in *S. aureus*, MgrA plays a role in the regulation of biofilm and virulence^85^, ^86^. A strain with a mutated *mgrA* showed a substantial decrease in virulence in a mouse infection model^87^. A unique cysteine residue (Cys12) is located at the interface of the protein dimer and can be oxidized by various ROS, which leads to the dissociation of MgrA from DNA^28^. A recent study revealed that MgrA also senses sulfane sulfur^23^. Sulfane sulfur modifies MgrA activity by forming Cys12 persulfide (Cys12‐SSH) (Figure 3), decreasing the binding of MgrA to its cognate DNA sites, and impacting the transcription of its regulated genes.

## THE OVERLAP AND DIFFERENCE BETWEEN ROS AND SULFANE SULFUR SENSED BY MarR REGULATORS

**Figure 4:**
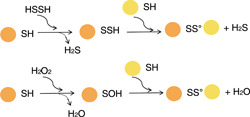
Schematic representations of the protein thiols that react with sulfane sulfur species or reactive oxygen species to produce disulfide bonds.

As reviewed earlier, all MarR regulators that sense sulfane sulfur also respond to ROS^24^, ^25^, ^26^. The similarity is likely due to their chemical properties^88^. Both S and O are chalcogens. Sulfane sulfur species and ROS (e.g., HSSH vs. H_2_O_2_) react with protein thiols to produce protein‐SSH and protein‐SOH, respectively. Both can rapidly react with a nearby protein thiol to produce a disulfide bond^89^ (Figure 4). The effects of protein‐SSH and protein‐SOH may be similar to the configuration changes of the MarR regulators, and both lead to the formation of protein disulfide bonds (Figure 4). Furthermore, the modification of proteins by sulfane sulfur may result in multiple sulfur links such as in FisR and SqrR^64^, ^65^, which is not possible by ROS modification.

## THE EVOLUTION ASPECT OF MarR REGULATORS

**Figure 5:**
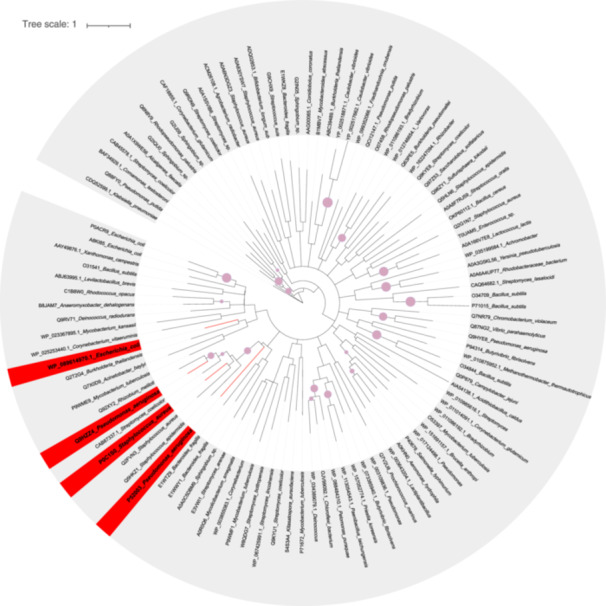
The phylogeny relationship of MarR sequences collected from published articles. Using “MarR” and “regulator” as keywords, we collected MarR protein sequences published in 180 SCI articles in the past 5 years. Cluster database at high identity with tolerance (CD‐HIT) was used to cluster them at a protein similarity threshold of <40%, and 103 MarR sequences were left. The 103 MarR family proteins were used to construct a maximum likelihood tree using IQ‐TREE with a bootstrap analysis of 1000 repeats. The MarR family proteins that have been reported to sense sulfane sulfur, including MgrA (Uniprot: P0C1S0) from *Staphylococcus aureus*, MexR (Uniprot: P52003) and OhrR (Uniprot: Q9HZZ4) from *Pseudomonas aeruginosa* PAO1, and MarR (WP_089614970.1) from *Escherichia coli*, are presented in red. The pink solid circles on the branches indicate that the bootstrap value is >50; that is, the larger the circle, the larger the value.

The initial modulator of MarR regulators is possibly sulfane sulfur. Most of the characterized MarR members can sense ROS^46^, ^90^, ^91^. Recently, the ROS‐sensing MarR family proteins have been shown to also sense sulfane sulfur^24^, ^25^, ^26^. This is in agreement with the similar chemistry of ROS and sulfane sulfur toward protein thiols^69^. As sulfur is traced back before the Great Oxidation Event, when O_2_ was generated by cyanobacteria, the ability to sense sulfane sulfur by the MarR members likely predates their ROS sensing. The long history of the MarR family correlates well with the wide distribution of MarR family proteins in sequenced bacterial genomes with approximately seven genes per genome^4^. Through evolution, MarR members have gained the ability to bind small organic modulators at the junction between the dimerization and DNA‐binding domains, even though they do not have a specific substrate‐binding domain^92^, ^93^. The cysteine residues involved in ROS and sulfur sensing may also be involved in metal sensing^25^, ^27^ and may have also evolved to involve other amino acid residues in metal sensing^59^. Phylogenetic analyses of the known MarR regulators revealed that MgrA, MexR, and OhrR are from the same clade, but MarR is from another clade (Figure 5). The results showed that the MarR family proteins with sulfur‐sensing ability are relatively randomly dispersed, suggesting that their sulfur‐sensing ability may not be recently acquired. Further characterization of MarR family proteins may lead to a better conclusion.

## CONCLUSION

MarR family proteins demonstrate restricted sequence conservation across lineages even though they are prominently present in various bacterial species. The variation may reflect the inherent versatility of the MarR family proteins that allow them to respond to various physiological and environmental signals. Recent studies have highlighted the critical roles of ROS sensing by MarR family regulators. MexR, MarR, OhrR, and MgrA are members of the MarR family. Although they control diverse cellular functions and respond to different inducers, they all sense sulfane sulfur (Figure 3). MexR, OhrR, and MgrA also sense ROS. Sulfane sulfur species have chemical properties similar to those of ROS (e.g., HSSH vs. H_2_O_2_). From an evolutionary perspective, the history of S on Earth is much longer than that of O. As an abundant element on ancient Earth, S might have played regulating roles in ancient microorganisms. Thus, it is not surprising that like ROS, sulfane sulfur is also an important modulator of MarR family regulators. Sulfane sulfur is a regular cellular component that undergoes changes with growth phases. This suggests its potential role as a universal signaling molecule in bacteria. The limited examples indicate that sulfane sulfur directly modifies MarR family regulators, which leads to diverse microbial behaviors; however, further efforts are required to establish that sulfane sulfur is a common inducer of the MarR family regulators. As sulfane sulfur is a regular cellular component, its variations during growth may be an intrinsic signal to confer bacteria to resist certain antibiotics without being induced by them.
